# Supplementary material for: The impact of the COVID-19 pandemic on Italian population-based cancer screening activities and test coverage: Results from national cross-sectional repeated surveys in 2020
Source: eLife. 2023 Feb 3;12:e81804. doi: 10.7554/eLife.81804 (PMC9934859; doi:10.7554/eLife.81804)
Supplement: Supplementary file 7. [file elife-81804-supp7.docx]

Supplementary table. Denominators (N) of Trends of the proportion of the screening target population who declared to have had a test in due time shown in figure 5

**Cervix**

| **Year** | **quarter** | **Women aged 25 to 64** | | | | | | | | |
| --- | --- | --- | --- | --- | --- | --- | --- | --- | --- | --- |
|  |  | **Total** | **North** | **Center** | **South and Island** | **Low education** | **High education** | **Many perceived economic difficulties** | **Some perceived economic difficulties** | **None perceived economic difficulties** |
| 2008 | Jan-08 | 3.308 | 1.827 | 723 | 758 | 1.406 | 1.899 | 477 | 1.398 | 1.426 |
| 2008 | Apr-08 | 3.717 | 1.925 | 774 | 1.018 | 1.594 | 2.121 | 525 | 1.615 | 1.561 |
| 2008 | Jul-08 | 2.940 | 1.440 | 681 | 819 | 1.244 | 1.693 | 436 | 1.265 | 1.231 |
| 2008 | Oct-08 | 4.166 | 2.000 | 1.097 | 1.069 | 1.745 | 2.418 | 559 | 1.744 | 1.849 |
| 2009 | Jan-09 | 4.297 | 2.170 | 978 | 1.149 | 1.773 | 2.521 | 567 | 1.810 | 1.907 |
| 2009 | Apr-09 | 4.476 | 2.014 | 1.384 | 1.078 | 1.839 | 2.635 | 621 | 1.852 | 1.989 |
| 2009 | Jul-09 | 3.034 | 1.523 | 796 | 715 | 1.261 | 1.773 | 353 | 1.256 | 1.417 |
| 2009 | Oct-09 | 3.914 | 2.039 | 909 | 966 | 1.597 | 2.314 | 469 | 1.552 | 1.877 |
| 2010 | Jan-10 | 3.622 | 1.964 | 920 | 738 | 1.467 | 2.153 | 426 | 1.432 | 1.752 |
| 2010 | Apr-10 | 3.996 | 2.063 | 982 | 951 | 1.568 | 2.427 | 516 | 1.633 | 1.841 |
| 2010 | Jul-10 | 3.028 | 1.515 | 729 | 784 | 1.156 | 1.870 | 326 | 1.225 | 1.472 |
| 2010 | Oct-10 | 4.156 | 2.058 | 1.036 | 1.062 | 1.650 | 2.506 | 479 | 1.703 | 1.963 |
| 2011 | Jan-11 | 3.732 | 1.900 | 887 | 945 | 1.469 | 2.262 | 429 | 1.534 | 1.765 |
| 2011 | Apr-11 | 4.014 | 1.922 | 983 | 1.109 | 1.521 | 2.489 | 486 | 1.656 | 1.866 |
| 2011 | Jul-11 | 3.057 | 1.469 | 805 | 783 | 1.214 | 1.837 | 431 | 1.242 | 1.375 |
| 2011 | Oct-11 | 4.103 | 2.056 | 1.003 | 1.044 | 1.535 | 2.561 | 578 | 1.770 | 1.748 |
| 2012 | Jan-12 | 3.871 | 1.898 | 865 | 1.108 | 1.412 | 2.456 | 580 | 1.686 | 1.595 |
| 2012 | Apr-12 | 3.849 | 1.927 | 944 | 978 | 1.452 | 2.390 | 581 | 1.655 | 1.605 |
| 2012 | Jul-12 | 3.126 | 1.473 | 753 | 900 | 1.098 | 2.017 | 492 | 1.339 | 1.284 |
| 2012 | Oct-12 | 4.422 | 2.104 | 1.001 | 1.317 | 1.659 | 2.761 | 705 | 1.998 | 1.710 |
| 2013 | Jan-13 | 4.692 | 1.977 | 1.577 | 1.138 | 1.729 | 2.962 | 740 | 2.081 | 1.867 |
| 2013 | Apr-13 | 4.410 | 1.822 | 1.444 | 1.144 | 1.623 | 2.785 | 691 | 1.943 | 1.773 |
| 2013 | Jul-13 | 2.948 | 1.384 | 718 | 846 | 1.048 | 1.893 | 484 | 1.236 | 1.219 |
| 2013 | Oct-13 | 4.206 | 1.912 | 1.084 | 1.210 | 1.539 | 2.661 | 663 | 1.866 | 1.666 |
| 2014 | Jan-14 | 3.564 | 1.790 | 840 | 934 | 1.235 | 2.326 | 582 | 1.554 | 1.424 |
| 2014 | Apr-14 | 3.743 | 1.759 | 957 | 1.027 | 1.309 | 2.427 | 558 | 1.609 | 1.561 |
| 2014 | Jul-14 | 2.931 | 1.484 | 770 | 677 | 1.040 | 1.890 | 454 | 1.235 | 1.222 |
| 2014 | Oct-14 | 3.943 | 1.775 | 1.027 | 1.141 | 1.335 | 2.606 | 613 | 1.718 | 1.605 |
| 2015 | Jan-15 | 3.828 | 1.715 | 900 | 1.213 | 1.342 | 2.483 | 527 | 1.590 | 1.702 |
| 2015 | Apr-15 | 3.633 | 1.656 | 925 | 1.052 | 1.256 | 2.377 | 532 | 1.557 | 1.532 |
| 2015 | Jul-15 | 2.764 | 1.353 | 656 | 755 | 890 | 1.873 | 384 | 1.162 | 1.215 |
| 2015 | Oct-15 | 3.878 | 1.687 | 1.018 | 1.173 | 1.302 | 2.574 | 537 | 1.587 | 1.747 |
| 2016 | Jan-16 | 3.566 | 1.811 | 758 | 997 | 1.153 | 2.411 | 456 | 1.438 | 1.656 |
| 2016 | Apr-16 | 3.481 | 1.638 | 777 | 1.066 | 1.111 | 2.369 | 458 | 1.433 | 1.564 |
| 2016 | Jul-16 | 2.772 | 1.286 | 782 | 704 | 867 | 1.905 | 339 | 1.078 | 1.339 |
| 2016 | Oct-16 | 3.985 | 1.658 | 1.178 | 1.149 | 1.188 | 2.794 | 450 | 1.636 | 1.874 |
| 2017 | Jan-17 | 3.163 | 1.407 | 988 | 768 | 1.041 | 2.120 | 346 | 1.286 | 1.521 |
| 2017 | Apr-17 | 3.309 | 1.526 | 721 | 1.062 | 1.059 | 2.248 | 430 | 1.331 | 1.543 |
| 2017 | Jul-17 | 2.383 | 1.105 | 566 | 712 | 719 | 1.663 | 274 | 983 | 1.124 |
| 2017 | Oct-17 | 3.837 | 1.502 | 1.045 | 1.290 | 1.170 | 2.664 | 430 | 1.557 | 1.846 |
| 2018 | Jan-18 | 2.815 | 1.192 | 686 | 937 | 852 | 1.955 | 325 | 1.126 | 1.359 |
| 2018 | Apr-18 | 3.257 | 1.350 | 795 | 1.112 | 962 | 2.295 | 345 | 1.297 | 1.605 |
| 2018 | Jul-18 | 2.567 | 1.056 | 698 | 813 | 753 | 1.812 | 291 | 979 | 1.280 |
| 2018 | Oct-18 | 3.838 | 1.445 | 1.129 | 1.264 | 1.139 | 2.696 | 369 | 1.485 | 1.964 |
| 2019 | Jan-19 | 3.073 | 1.216 | 850 | 1.007 | 892 | 2.178 | 271 | 1.137 | 1.649 |
| 2019 | Apr-19 | 3.324 | 1.356 | 842 | 1.126 | 987 | 2.333 | 322 | 1.275 | 1.707 |
| 2019 | Jul-19 | 2.789 | 1.258 | 694 | 837 | 765 | 2.022 | 254 | 1.004 | 1.523 |
| 2019 | Oct-19 | 3.700 | 1.344 | 1.017 | 1.339 | 1.041 | 2.657 | 335 | 1.378 | 1.975 |
| 2020 | Jan-20 | 2.132 | 743 | 597 | 792 | 568 | 1.560 | 200 | 793 | 1.129 |
| 2020 | Apr-20 | 1.429 | 343 | 465 | 621 | 343 | 1.085 | 114 | 511 | 801 |
| 2020 | Jul-20 | 1.280 | 287 | 386 | 607 | 331 | 947 | 122 | 454 | 704 |
| 2020 | Oct-20 | 2.193 | 550 | 398 | 1.245 | 560 | 1.627 | 192 | 842 | 1.151 |

**Breast**

| **Year** | **quarter** | **Women aged 50 to 69** | | | | | | | | |
| --- | --- | --- | --- | --- | --- | --- | --- | --- | --- | --- |
|  |  | **Totale** | **North** | **Center** | **South and Island** | **Low education** | **High education** | **Many perceived economic difficulties** | **Some perceived economic difficulties** | **None perceived economic difficulties** |
| 2008 | Jan-08 | 1.558 | 907 | 329 | 322 | 1.051 | 506 | 247 | 644 | 664 |
| 2008 | Apr-08 | 1.729 | 943 | 372 | 414 | 1.158 | 570 | 302 | 714 | 705 |
| 2008 | Jul-08 | 1.378 | 676 | 320 | 382 | 907 | 471 | 229 | 599 | 547 |
| 2008 | Oct-08 | 2.043 | 969 | 596 | 478 | 1.364 | 677 | 305 | 863 | 867 |
| 2009 | Jan-09 | 1.953 | 1.052 | 453 | 448 | 1.257 | 694 | 268 | 804 | 874 |
| 2009 | Apr-09 | 2.125 | 944 | 706 | 475 | 1.382 | 740 | 313 | 866 | 937 |
| 2009 | Jul-09 | 1.466 | 746 | 393 | 327 | 973 | 493 | 188 | 626 | 650 |
| 2009 | Oct-09 | 1.828 | 936 | 448 | 444 | 1.167 | 659 | 253 | 731 | 834 |
| 2010 | Jan-10 | 1.671 | 925 | 406 | 340 | 1.068 | 600 | 197 | 640 | 825 |
| 2010 | Apr-10 | 1.827 | 963 | 465 | 399 | 1.144 | 682 | 250 | 768 | 807 |
| 2010 | Jul-10 | 1.418 | 731 | 339 | 348 | 876 | 540 | 192 | 570 | 653 |
| 2010 | Oct-10 | 1.933 | 967 | 504 | 462 | 1.194 | 738 | 252 | 795 | 876 |
| 2011 | Jan-11 | 1.734 | 884 | 413 | 437 | 1.096 | 638 | 215 | 714 | 803 |
| 2011 | Apr-11 | 1.848 | 918 | 458 | 472 | 1.092 | 754 | 256 | 748 | 841 |
| 2011 | Jul-11 | 1.452 | 687 | 406 | 359 | 896 | 554 | 219 | 607 | 623 |
| 2011 | Oct-11 | 1.985 | 989 | 470 | 526 | 1.152 | 828 | 303 | 849 | 829 |
| 2012 | Jan-12 | 1.782 | 903 | 419 | 460 | 1.032 | 748 | 259 | 768 | 751 |
| 2012 | Apr-12 | 1.819 | 934 | 438 | 447 | 1.038 | 775 | 260 | 753 | 800 |
| 2012 | Jul-12 | 1.467 | 690 | 372 | 405 | 852 | 605 | 238 | 653 | 569 |
| 2012 | Oct-12 | 2.166 | 1.050 | 491 | 625 | 1.305 | 861 | 388 | 931 | 845 |
| 2013 | Jan-13 | 2.233 | 945 | 781 | 507 | 1.299 | 934 | 363 | 981 | 888 |
| 2013 | Apr-13 | 2.139 | 885 | 715 | 539 | 1.213 | 925 | 349 | 915 | 871 |
| 2013 | Jul-13 | 1.395 | 669 | 349 | 377 | 751 | 641 | 217 | 560 | 612 |
| 2013 | Oct-13 | 2.059 | 958 | 547 | 554 | 1.205 | 853 | 319 | 903 | 833 |
| 2014 | Jan-14 | 1.708 | 874 | 410 | 424 | 971 | 736 | 277 | 767 | 662 |
| 2014 | Apr-14 | 1.848 | 884 | 470 | 494 | 1.037 | 808 | 243 | 803 | 795 |
| 2014 | Jul-14 | 1.446 | 748 | 383 | 315 | 813 | 632 | 221 | 605 | 606 |
| 2014 | Oct-14 | 1.938 | 934 | 485 | 519 | 1.035 | 901 | 267 | 856 | 810 |
| 2015 | Jan-15 | 1.924 | 917 | 447 | 560 | 1.036 | 888 | 283 | 756 | 882 |
| 2015 | Apr-15 | 1.851 | 861 | 490 | 500 | 1.013 | 838 | 275 | 790 | 776 |
| 2015 | Jul-15 | 1.408 | 704 | 348 | 356 | 721 | 687 | 202 | 583 | 621 |
| 2015 | Oct-15 | 2.000 | 902 | 519 | 579 | 1.077 | 922 | 324 | 802 | 871 |
| 2016 | Jan-16 | 1.808 | 963 | 380 | 465 | 954 | 853 | 240 | 740 | 814 |
| 2016 | Apr-16 | 1.759 | 869 | 393 | 497 | 962 | 796 | 242 | 724 | 780 |
| 2016 | Jul-16 | 1.407 | 660 | 394 | 353 | 655 | 752 | 174 | 545 | 673 |
| 2016 | Oct-16 | 2.061 | 859 | 641 | 561 | 1.017 | 1.043 | 248 | 822 | 976 |
| 2017 | Jan-17 | 1.566 | 765 | 468 | 333 | 779 | 786 | 179 | 615 | 766 |
| 2017 | Apr-17 | 1.703 | 797 | 368 | 538 | 846 | 857 | 227 | 688 | 785 |
| 2017 | Jul-17 | 1.256 | 586 | 294 | 376 | 616 | 640 | 164 | 530 | 562 |
| 2017 | Oct-17 | 2.007 | 785 | 533 | 689 | 989 | 1.017 | 254 | 824 | 928 |
| 2018 | Jan-18 | 1.422 | 638 | 334 | 450 | 667 | 749 | 183 | 533 | 703 |
| 2018 | Apr-18 | 1.658 | 745 | 372 | 541 | 785 | 872 | 182 | 651 | 822 |
| 2018 | Jul-18 | 1.336 | 557 | 370 | 409 | 649 | 687 | 160 | 489 | 673 |
| 2018 | Oct-18 | 2.004 | 782 | 596 | 626 | 939 | 1.062 | 193 | 798 | 994 |
| 2019 | Jan-19 | 1.601 | 695 | 434 | 472 | 744 | 856 | 152 | 586 | 860 |
| 2019 | Apr-19 | 1.747 | 753 | 430 | 564 | 805 | 941 | 167 | 677 | 889 |
| 2019 | Jul-19 | 1.486 | 674 | 387 | 425 | 662 | 823 | 122 | 549 | 811 |
| 2019 | Oct-19 | 1.882 | 735 | 513 | 634 | 841 | 1.040 | 189 | 677 | 1.008 |
| 2020 | Jan-20 | 1.146 | 408 | 339 | 399 | 492 | 651 | 118 | 421 | 600 |
| 2020 | Apr-20 | 752 | 174 | 252 | 326 | 295 | 456 | 55 | 259 | 437 |
| 2020 | Jul-20 | 692 | 177 | 218 | 297 | 305 | 385 | 72 | 251 | 369 |
| 2020 | Oct-20 | 1.096 | 300 | 206 | 590 | 474 | 620 | 97 | 424 | 571 |

**Colorectal**

| **Year** | **quarter** | **People aged 50 to 69** | | | | | | | | |
| --- | --- | --- | --- | --- | --- | --- | --- | --- | --- | --- |
|  |  | **Totale** | **North** | **Center** | **South and Island** | **Low education** | **High education** | **Many perceived economic difficulties** | **Some perceived economic difficulties** | **None perceived economic difficulties** |
| 2010 | Jan-10 | 2.290 | 1.335 | 530 | 425 | 1.402 | 885 | 244 | 860 | 1.177 |
| 2010 | Apr-10 | 3.344 | 1.726 | 864 | 754 | 2.053 | 1.289 | 421 | 1.364 | 1.551 |
| 2010 | Jul-10 | 2.574 | 1.242 | 660 | 672 | 1.527 | 1.044 | 332 | 995 | 1.241 |
| 2010 | Oct-10 | 3.607 | 1.731 | 951 | 925 | 2.171 | 1.436 | 423 | 1.433 | 1.741 |
| 2011 | Jan-11 | 3.292 | 1.644 | 779 | 869 | 1.983 | 1.309 | 380 | 1.296 | 1.614 |
| 2011 | Apr-11 | 3.466 | 1.642 | 894 | 930 | 2.021 | 1.443 | 440 | 1.360 | 1.660 |
| 2011 | Jul-11 | 2.736 | 1.248 | 781 | 707 | 1.635 | 1.098 | 358 | 1.091 | 1.282 |
| 2011 | Oct-11 | 3.617 | 1.749 | 897 | 971 | 2.079 | 1.530 | 539 | 1.446 | 1.625 |
| 2012 | Jan-12 | 3.385 | 1.613 | 812 | 960 | 1.955 | 1.429 | 477 | 1.423 | 1.478 |
| 2012 | Apr-12 | 3.382 | 1.661 | 844 | 877 | 1.880 | 1.490 | 483 | 1.397 | 1.490 |
| 2012 | Jul-12 | 2.762 | 1.258 | 692 | 812 | 1.581 | 1.166 | 429 | 1.177 | 1.144 |
| 2012 | Oct-12 | 3.983 | 1.817 | 964 | 1.202 | 2.322 | 1.660 | 642 | 1.688 | 1.646 |
| 2013 | Jan-13 | 4.226 | 1.672 | 1.487 | 1.067 | 2.348 | 1.878 | 650 | 1.807 | 1.765 |
| 2013 | Apr-13 | 4.089 | 1.612 | 1.414 | 1.063 | 2.288 | 1.799 | 656 | 1.684 | 1.741 |
| 2013 | Jul-13 | 2.555 | 1.153 | 668 | 734 | 1.378 | 1.174 | 384 | 1.024 | 1.141 |
| 2013 | Oct-13 | 3.782 | 1.676 | 1.027 | 1.079 | 2.109 | 1.671 | 569 | 1.604 | 1.601 |
| 2014 | Jan-14 | 3.172 | 1.545 | 779 | 848 | 1.768 | 1.402 | 504 | 1.339 | 1.324 |
| 2014 | Apr-14 | 3.434 | 1.576 | 916 | 942 | 1.855 | 1.574 | 486 | 1.441 | 1.498 |
| 2014 | Jul-14 | 2.668 | 1.310 | 730 | 628 | 1.449 | 1.217 | 397 | 1.063 | 1.184 |
| 2014 | Oct-14 | 3.633 | 1.668 | 954 | 1.011 | 1.932 | 1.696 | 522 | 1.526 | 1.578 |
| 2015 | Jan-15 | 3.480 | 1.568 | 846 | 1.066 | 1.852 | 1.627 | 509 | 1.331 | 1.634 |
| 2015 | Apr-15 | 3.392 | 1.504 | 915 | 973 | 1.761 | 1.631 | 462 | 1.429 | 1.485 |
| 2015 | Jul-15 | 2.595 | 1.221 | 663 | 711 | 1.263 | 1.331 | 359 | 994 | 1.237 |
| 2015 | Oct-15 | 3.724 | 1.618 | 1.004 | 1.102 | 1.939 | 1.783 | 545 | 1.445 | 1.731 |
| 2016 | Jan-16 | 3.281 | 1.639 | 725 | 917 | 1.664 | 1.615 | 427 | 1.250 | 1.585 |
| 2016 | Apr-16 | 3.188 | 1.490 | 726 | 972 | 1.651 | 1.535 | 419 | 1.252 | 1.495 |
| 2016 | Jul-16 | 2.530 | 1.162 | 693 | 675 | 1.229 | 1.300 | 313 | 948 | 1.240 |
| 2016 | Oct-16 | 3.767 | 1.494 | 1.199 | 1.074 | 1.844 | 1.923 | 452 | 1.482 | 1.812 |
| 2017 | Jan-17 | 2.931 | 1.311 | 958 | 662 | 1.420 | 1.510 | 342 | 1.096 | 1.481 |
| 2017 | Apr-17 | 3.101 | 1.350 | 702 | 1.049 | 1.555 | 1.546 | 392 | 1.205 | 1.499 |
| 2017 | Jul-17 | 2.324 | 1.027 | 539 | 758 | 1.143 | 1.180 | 293 | 894 | 1.135 |
| 2017 | Oct-17 | 3.702 | 1.377 | 973 | 1.352 | 1.821 | 1.880 | 464 | 1.416 | 1.818 |
| 2018 | Jan-18 | 2.623 | 1.077 | 659 | 887 | 1.245 | 1.369 | 316 | 956 | 1.343 |
| 2018 | Apr-18 | 3.031 | 1.280 | 677 | 1.074 | 1.451 | 1.579 | 318 | 1.136 | 1.572 |
| 2018 | Jul-18 | 2.430 | 957 | 701 | 772 | 1.178 | 1.252 | 283 | 870 | 1.255 |
| 2018 | Oct-18 | 3.833 | 1.358 | 1.178 | 1.297 | 1.786 | 2.043 | 406 | 1.407 | 1.983 |
| 2019 | Jan-19 | 2.878 | 1.121 | 832 | 925 | 1.361 | 1.516 | 284 | 1.005 | 1.585 |
| 2019 | Apr-19 | 3.150 | 1.295 | 799 | 1.056 | 1.463 | 1.687 | 288 | 1.122 | 1.716 |
| 2019 | Jul-19 | 2.688 | 1.135 | 739 | 814 | 1.211 | 1.475 | 231 | 940 | 1.505 |
| 2019 | Oct-19 | 3.562 | 1.294 | 1.002 | 1.266 | 1.618 | 1.943 | 347 | 1.258 | 1.943 |
| 2020 | Jan-20 | 2.031 | 712 | 633 | 686 | 875 | 1.154 | 191 | 684 | 1.150 |
| 2020 | Apr-20 | 1.360 | 317 | 451 | 592 | 550 | 809 | 108 | 450 | 800 |
| 2020 | Jul-20 | 1.318 | 293 | 430 | 595 | 580 | 736 | 125 | 441 | 751 |
| 2020 | Oct-20 | 2.062 | 521 | 398 | 1.143 | 900 | 1.160 | 168 | 762 | 1.126 |
